# HDO production from [^2^H_7_]glucose Quantitatively Identifies Warburg Metabolism

**DOI:** 10.1038/s41598-020-65839-8

**Published:** 2020-06-01

**Authors:** Rohit Mahar, Patrick L. Donabedian, Matthew E. Merritt

**Affiliations:** Department of Biochemistry and Molecular Biology, College of Medicine, University of Florida, Gainesville, FL 32610 USA

**Keywords:** Metabolomics, Cancer imaging, Cancer metabolism

## Abstract

Increased glucose uptake and aerobic glycolysis are striking features of many cancers. These features have led to many techniques for screening and diagnosis, but many are expensive, less feasible or have harmful side-effects. Here, we report a sensitive ^1^H/^2^H NMR method to measure the kinetics of lactate isotopomer and HDO production using a deuterated tracer. To test this hypothesis, HUH-7 hepatocellular carcinoma and AML12 normal hepatocytes were incubated with [^2^H_7_]glucose. ^1^H/^2^H NMR data were recorded for cell media as a function of incubation time. The efflux rate of lactate-CH_3_, lactate-CH_2_D and lactate-CHD_2_ was calculated as 0.0033, 0.0071, and 0.0.012 µmol/10^6^cells/min respectively. Differential production of lactate isotopomers was due to deuterium loss during glycolysis. Glucose uptake and HDO production by HUH-7 cells showed a strong correlation, indicating that monitoring the HDO production could be a diagnostic feature in cancers. Deuterium mass balance of [^2^H_7_]glucose uptake to ^2^H-lactate and HDO production is quantitatively matched, suggesting increasing HDO signal could be used to diagnose Warburg (cancer) metabolism. Measuring the kinetics of lactate isotopomer and HDO production by ^1^H and ^2^H MR respectively are highly sensitive. Increased T_1_ of ^2^H-lactate isotopomers indicates inversion/saturation recovery methods may be a simple means of generating metabolism-based contrast.

## Introduction

Central carbon metabolism plays an important role in the origin or progression of many diseases, including cancers, neurodegenerative diseases, diabetes, and nonalcoholic fatty liver disease (NAFLD)^1–3^. Metabolic reprogramming is one of the key features of cancer cell metabolism. Many cancer cells consume tremendous amounts of glucose and generate ATP *via* glycolysis even under normoxic conditions (the Warburg effect)^4^. Many theories have been promulgated to explain this observation, ranging from impaired mitochondrial function as the primary causative agent of cancer^5^, to a trade-off of inefficient energy metabolism for faster incorporation of carbon into biomass^6^, to a double selective advantage of hypoxia resistance and generation of local acidosis^7^. Molecular imaging techniques capable of identifying such differences have become very important for cancer diagnosis, disease progression, and treatment. Glucose tracers have been widely utilized for accessing the metabolic conversion of glucose to lactate predominantly *via* glycolysis. Metabolic imaging of cancer, which relies on unique physiologic hallmarks^8^, is sensitive but its widespread use is mainly limited to nuclear medicine. In the clinic, the glucose analog [^18^F]2-fluoro-2-deoxy-glucose (^18^FDG) is used for positron emission tomography (PET) high-resolution maps of glucose uptake, but it cannot report differences in flux aside from glucose uptake, and is associated with doses of ionizing radiation that carry an unknown, but real, risk of secondary cancer^9,10^. These tests are therefore limited to patients with known or suspected malignancy^11^. Also, due to limited lifetime radiation burden for a patient, ^18^FDG is less suitable for repetitive scanning for disease progression^12^. CEST MRI was developed to overcome some of the limitations of PET for glucose imaging. CEST detects glucose uptake indirectly by saturation transfer from the exchangeable protons of glucose to water, which enhances sensitivity relative to direct detection, but the technique is hampered by water exchange rates at physiological pH^13,14^. Dissolution dynamic nuclear polarization (dDNP) was developed to enhance SNR in ^13^C MR *via* polarization transfer from an electron source to nuclei, resulting in >10,000 times signal enhancement in the MRI signal^15,16^. However, dDNP is limited to a small set of molecules having the long relaxation times necessary to measure the kinetics before polarized signal is lost. Many important metabolites, *i.e*. glucose, have short relaxation times, and are difficult to image using dDNP methods^17^. In addition, lactate pool size convolves with lactate production rates to make fitting of dDNP dynamics subject to multiple preconditions for successful modeling^18^. Analysis of lactate isotopomers has been carried out using ^13^C labeled glucose and spin editing, but the ^13^C enrichment is not easily determined in some cases due to overlap with fatty acid (FA) resonances^19^. Due to the multiplicities from nuclear spin–spin couplings of the ^1^H-^13^C in lactate ^13^C-isotopomers, ^1^H-NMR spectra become complex. Direct ^13^C acquisition provides a straightforward method for isotopomer analysis but requires long data acquisition time^20^.

Deuterium (^2^H) magnetic resonance imaging (DMI), a new method for assessing metabolic flux in functioning tissues, cell culture, and *in vivo*, provides the requisite chemical, spatial and temporal resolution needed for cancer detection^21^. Nonradioactive ^2^H-labeled substrates can be utilized to observe the downstream metabolites, due to very low natural abundance of ^2^H (0.0115%), leading to high tracer specificity. Administration of deuterium-labeled substrates at tracer levels (<200 mg/kg ^2^H) is well established as safe^22–24^. *In vivo* ^2^H MR was investigated in the late 1980’s^25–28^, but has recently enjoyed a resurgence due to improved technology and the accumulation of decades of basic research on cancer metabolic phenotypes^29^. Previous studies using ^2^H MRI to image cancer metabolism relied on differential production of TCA cycle intermediates and lactate^21^. Deuterated tracers are inexpensive and straightforward to administer, but ^2^H MR sensitivity is low. Indeed, the experiment is only tractable because the quadrupole of the ^2^H nucleus makes its T_1_ short, facilitating very short recycle time (Tr). To cope with low sensitivity of ^2^H-NMR experiment for less abundant metabolites, more sensitive experiments and methodology need to be designed. We reasoned that enrichment of ^2^H_2_O (*i.e*. HDO production) after administration of deuterium-labeled glucose would provide a sensitive marker of glucose utilization by tumor cells *in vivo*, with a possibility for a simplified imaging strategy.

**Figure 1:**
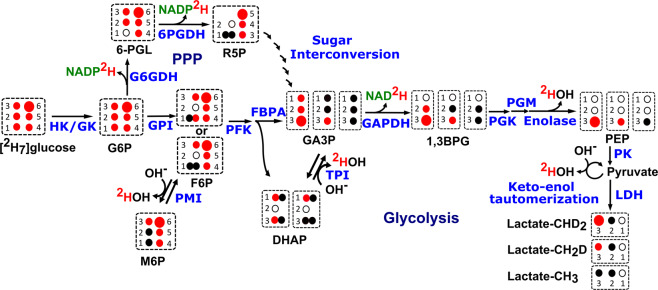
Lactate isotopomer and HDO (or ^2^HOH) production from [^2^H_7_]glucose: Schematic representation of lactate isotopomer and HDO production from [^2^H_7_]glucose during glycolysis and interconversion of F6P and M6P. Deuterium (^2^H) loss has been shown in the form of ^2^HOH, NADP^2^H and NAD^2^H during glycolysis and the pentose phosphate pathway. Large and small red filled circles represent 2 and 1 deuterium atoms respectively, black filled circles represent hydrogen atoms, and black empty circles represent quaternary carbons. Abbreviations are as follows: HK; hexokinase, GK; glucokinase, G6P; glucose-6-phosphate, F6P; fructose-6-phosphate, M6P; mannose-6-phosphate, GPI; glucose-6-phosphate isomerase, PFK; Phosphofructokinase, FBPA; fructose bisphosphate aldolase (or aldolase), G6PDH; glucose-6-phosphate dehydrogenase, PGL; phosphoglucono-lactone, 6PGDH; 6-phosphogluconate dehydrogenase, PMI; phosphomannoseisomerase, R5P; ribulose 5-phosphate, TPI; triose phosphate isomerase, DHAP; dihydroxy acetone phosphate, GA3P; glyceraldehyde 3-phosphate, GAPDH; glyceraldehyde phosphate dehydrogenase, PGK; phosphoglycerate kinase, PGM; phosphoglyceromutase, PEP; phospho-enol pyruvate, PK; pyruvate kinase, and LDH; lactate dehydrogenase. (Note: unlabeled lactate can be produced due to the deuterium loss from the deuterated precursors via TPI and by keto-enol tautomerization).

In this paper we have used highly sensitive ^1^H-NMR to measure the production of lactate isotopomers and ^2^H-NMR to measure HDO production and residual [^2^H_7_]glucose (eventually cellular glucose uptake) in the cell culture medium. The fate of the ^2^H tracer for lactate isotopomers, HDO production, and loss of ^2^H atoms from [^2^H_7_]glucose during glycolysis, sugar isomerization and pentose phosphate pathway (PPP) can be mapped using Fig. 1. Although deuterium *J*-coupled satellites are not well distinguished in ^1^H-NMR spectra due to the very low coupling constant of ^2^*J*_*H-D*_, ^2^H-decoupling and difference spectra can overcome these limitations^21^. Utilizing the small isotope shifts of mono-deuterated and di-deuterated lactate in the ^1^H-NMR spectrum, lactate-CH_2_D and lactate-CHD_2_ resonances are resolved, enabling a quantitative accounting of ^2^H disposal in this glycolytic end product. The estimates of HDO production rate combined with the production of the lactate isotopomers results in a complete accounting of the [^2^H_7_]glucose metabolism. An HUH-7 hepatocellular carcinoma cell line showed significantly increased HDO production versus a control AML12 cell line. The strong correlation between cellular glucose uptake and HDO production suggests HDO imaging is a promising approach for metabolic imaging of cancer that does not depend on exposure to radioactive isotopes.

## Results

### ^1^H-NMR spectra of the cell culture media

**Figure 2:**
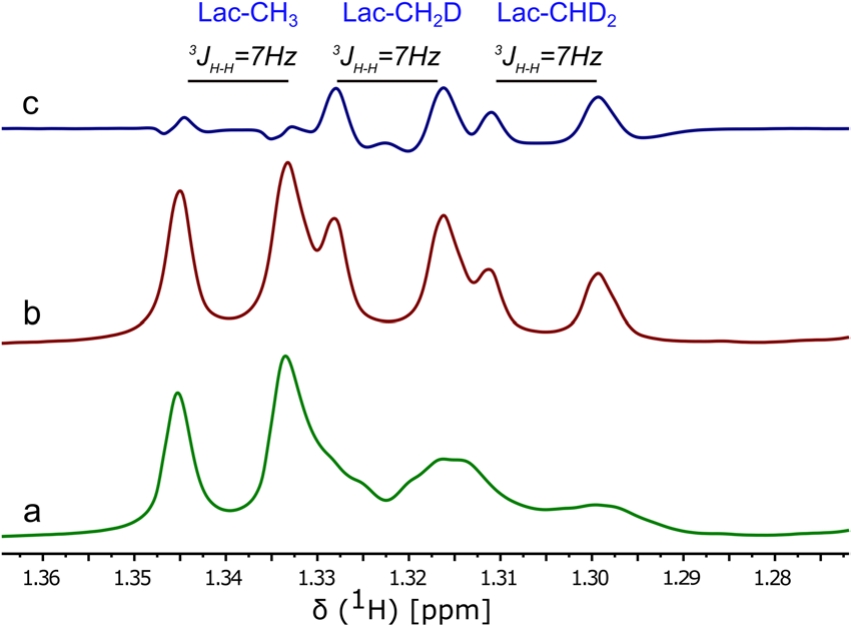
^2^H-decoupling off and on ^1^H-NMR spectra of lactate isotopomers: Representative expanded ^1^H-NMR of the lactate methyl group in the cell medium of the HUH-7 cancer cells incubated with 5.5 mM [^2^H_7_]glucose. (**a**) ^2^H-decoupling off, ^1^H-NMR spectra (**b**) ^2^H-decoupling on, ^1^H-NMR spectra and (**c**) the difference spectrum of the ^2^H-decoupling on and off ^1^H-NMR spectra. The resolved *J*-couplings arise from the hydrogen at the C2 position of lactate. The shifts in the methyl resonance position are caused by the isotope effect, with addition of ^2^H causing a shift to lower frequencies. The broadening of the spectrum in (**a**) arises from unresolved ^1^H-^2^H *J*-couplings. (Note: expanded spectral region was taken from the ^1^H-NMR data of 5 hour incubation time point of HUH-7 cells).

**Figure 3:**
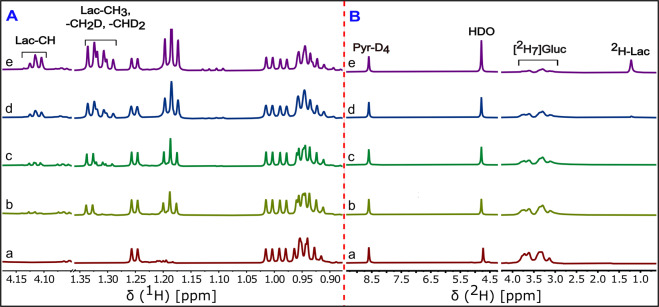
^1^H and ^2^H NMR spectra of cell media at different incubation time points of the HUH-7 cancer cells: Expanded stacked plot of the {^2^H}^1^H-NMR (left, A panel) and {^1^H}^2^H-NMR (right, B panel) spectra of the HUH-7 cell line incubated with 5.5 mM [^2^H_7_]glucose. Cell media withdrawn at (a) 0 min, (b) 20 min, (c) 1 h, (d) 2 h and (e) 5 h incubation time points from cultures of HUH-7 cancer cells. Labeling in the (e) spectrum of panel A showing lactate-CH and lactate-CH_3_, lactate-CH_2_D and lactate-CHD_2_ isotopomer shifts due to the ^2^H incorporation at the methyl position. In panel B, labeling on each peak in the (e) spectrum showing the resonances arise from the pyrazine-D_4_, HDO, residual [^2^H_7_]glucose, and ^2^H-lactate. (Note: the intensity scale of glucose and lactate region is 10x increased).

^1^H-NMR spectra with and without ^2^H-decoupling were recorded for cell media sampled from 0 to 5 hours. NMR spectra from the 5 hour time point show pronounced isotope shifts for lactate-CH_2_D and lactate-CHD_2_ isotopomers (Fig. 2a,b). Methyl signals of the deuterium-enriched lactate appear broad in the ^2^H-decoupling-off ^1^H-NMR spectrum, whereas methyl signals appear as sharp doublets in ^1^H-NMR spectrum acquired with ^2^H-decoupling on during the acquisition. Each replacement of a methyl proton with a deuteron introduces an isotope shift in the ^1^H spectrum of ~10 Hz. The difference spectrum of lactate-CH_2_D and lactate-CHD_2_ show the (^3^*J*_*H-H*_) of ~7.00 Hz clearly. The lactate-CH_3_ doublet at 1.34 ppm shows a very small antiphase doublet in the subtraction spectra due to small differences in peak position between blocks prior to subtraction. The metabolic scheme shown in Fig. 1 indicates the formation of mono-deuterated and di-deuterated lactate isotopomers from [^2^H_7_]glucose, clearly observed in the {^2^H}^1^H spectrum (Fig. 2b). The stacked plot of the {^2^H}^1^H-NMR spectra of cell media samples as a function of time shows progressive ^2^H enrichment of lactate (Fig. 3A). Intensity of ^1^H-NMR signals for all of the lactate isotopomers increases with incubation time. A summary of lactate enrichments is included in Table 1.

**Table 1:** Deuterium mass balance between number of µmol of ^2^H atoms of consumed [^2^H_7_]glucose and number of µmol of ^2^H atoms incorporated into the ^2^H-lactate & HDO by HUH-7 cancer cells and AML12 normal hepatic cells at 5 hour time points.

Cell lines	HUH-7		AML12	
	Concentration (µmol/L)	Number of µmol of ^2^H	Concentration (µmol/L)	Number of µmol of ^2^H
Consumed [^2^H_7_]glucose	3810.00 ± 485.83	26670.00 ± 3400.83	682.17 ± 108.45	4775.20 ± 438.32
Lactate-CHD_2_	2442.47	4884.9	353.07	706.14
Lactate-CH_2_D	1520.0	1520.0	229.26	229.26
HDO	18220.00 ± 3002.24	18220.00 ± 3002.24	3394.37 ± 705.14	3394.37 ± 705.14
µmol of ^2^H in ^2^H-lactate & HDO		24625.00		4329.77

HUH-7 consumed high amounts of [^2^H_7_]glucose, which eventually enriched the HDO and deuterated lactate isotopomers. AML12 cell lines did not produce a quantitative accounting for ^2^H label, likely due to oxidative flux and unmeasured labeling of TCA cycle intermediates. (Note: [^2^H_7_]glucose and HDO were measured from ^2^H-NMR data and ^2^H-lactate (lactate-CHD_2_ and lactate-CH_2_D) was measured from ^1^H-NMR data of 5 hours incubation time point samples of AML12 and HUH-7 cells. Data is represented as mean ± SEM for [^2^H_7_]glucose and HDO).

### ^2^H-NMR spectra of cell culture media

Proton-decoupled ^2^H-NMR spectra were recorded for each of the withdrawn cell media as a function of time for samples from HUH-7 and AML12 cell lines. HDO signal intensity increases with incubation time period whereas [^2^H_7_]glucose decreases rapidly for HUH-7 cells as indicated by the stacked plot of ^2^H-NMR spectra (Fig. 3, Panel B). The stacked plot of ^2^H-NMR spectra of AML12 cell media samples at different time points indicated slow increase of HDO and slow decrease of [^2^H_7_]glucose (see Supplementary Fig. S1). Qualitatively both stacked plots demonstrated that the cellular [^2^H_7_]glucose uptake and HDO production rate is very high in HUH-7 cancer cells compared to AML12 cells, reflecting the presence of Warburg metabolism.

### Longitudinal relaxation time (T_1_) measurement of the compounds

Once the lactate isotopomers were identified, it would be expected to observe differential changes in ^1^H T_1_ due to a loss of ^1^H dipolar relaxation. We performed T_1_ inversion recovery experiments in the ^2^H-decoupling on mode to calculate the T_1_ relaxation time of the pyrazine and lactate isotopomers (see Supplementary Fig. S2). A and B marks on the uppermost spectrum represent the pyrazine standard and methyl signals of the lactate isotopomers respectively. Integral areas of each of the lactate isotopomers and pyrazine standard were extracted to calculate the T_1_ relaxation times. Data was fit to the standard T_1_ equation, M_z_(t) = M_0_ (1–2xe^− τ/T1^), to extract the T_1_ s. Supplementary Figure S3 shows fitting curves for T_1_ measurement and T_1_ values of lactate-CH_3_, lactate-CH_2_D and lactate-CHD_2,_ are 1.85, 2.49 and 4.21 s respectively. T_1_ of the pyrazine standard was found to be 6.45 s. T_1_ relaxation time of the HDO and pyrazine-D_4_ standard were calculated using the inversion recovery ^2^H-NMR data (Supplementary Fig. S4) and found to be 0.49 and 0.45 s respectively. Supplementary Figure S6 includes the T_1_ measurement and T_1_ values of HDO and pyrazine-D_4_. This data was used to make a T_1_ correction to the concentrations of the lactate isotopomers.

### Quantification of the lactate isotopomers

**Figure 4:**
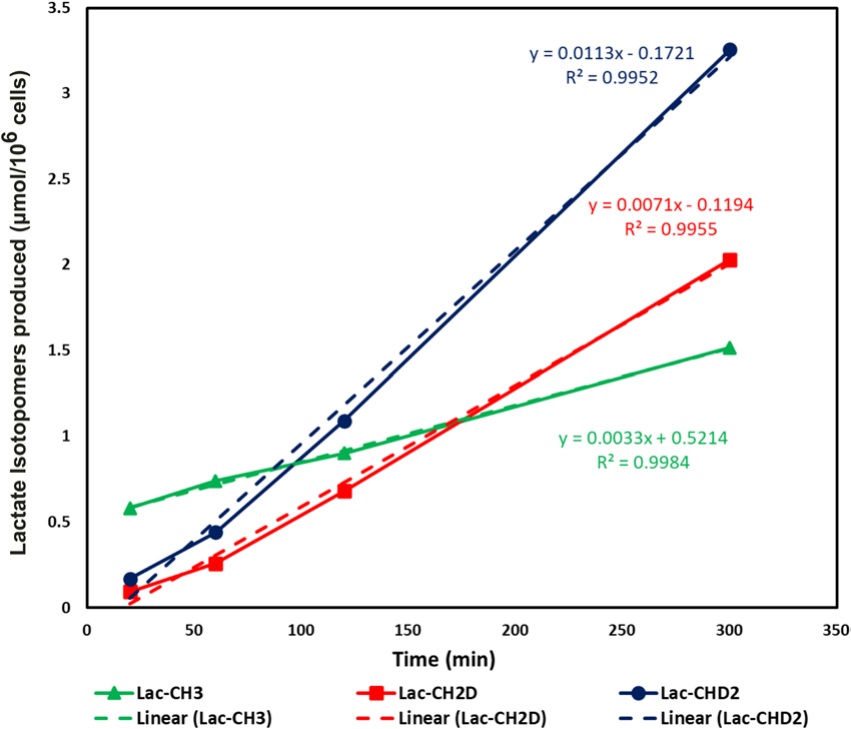
Linearity graph of the lactate isotopomers effluxed from HUH-7 cells into the cell media. Green, red, and blue curves demonstrating the relative rate of production of the Lac-CH_3_, Lac-CH_2_D and Lac-CHD_2_ with respect to the incubation time. Linear fits (dotted lines) were suitable models of lactate efflux from the cells. Even after switching media, unlabeled lactate continues to be produced. The concentrations are plotted and are derived from data normalized for differential T_1_s and the number of hydrogens leading to the resonances. (Note: Concentration of lactate isotopomers were measured from the {^2^H}^1^H-NMR spectrum of each sample withdrawn at 20 min, 1 h, 2 h and 5 h incubation time periods of HUH-7 cells).

**Figure 5:**
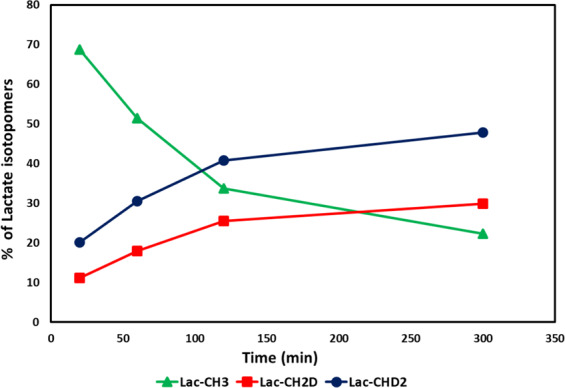
Relative pool size contribution of lactate isotopomers: Percent contribution of the lactate isotopomers to the lactate efflux pool size from the HUH-7 cells into the cell culture medium. Contribution of the Lac-CH_3_ decreases whereas Lac-CH_2_D and Lac-CHD_2_ increases. Fully deuterated lactate cannot be produced using [^2^H_7_]glucose. (Note: Percent contribution of the lactate isotopomers were measured from the concentration of lactate isotopomers in each sample withdrawn at 20 min, 1 h, 2 h and 5 h incubation time periods of HUH-7 cells).

Pyrazine was used as an internal standard to quantify each of the lactate isotopomers. Peak areas of the isotopomers were extracted from each of the {^2^H}^1^H-NMR spectra of the cell culture media at different incubation times. After correction for T_1_ and for the number of ^1^Hs contributing to each resonance, the concentration of the isotopomers was plotted (Fig. 4). All lactate isotopomers in the media increase in concentration with incubation time period. Linearity for lactate isotopomer production and efflux from HUH-7 cells to culture medium is excellent with R^2^ of 0.99. Initially, fully protonated lactate is primarily produced, subsequently switching to deuterated versions as more [^2^H_7_]glucose is consumed (Fig. 5). At the 5 hour time point, the measured lactate-CHD_2_ isotopomer was 1.6 fold higher in concentration than the -CH_2_D isotopomer.

### Quantification of the HDO and residual [^2^H_7_]glucose

**Figure 6:**
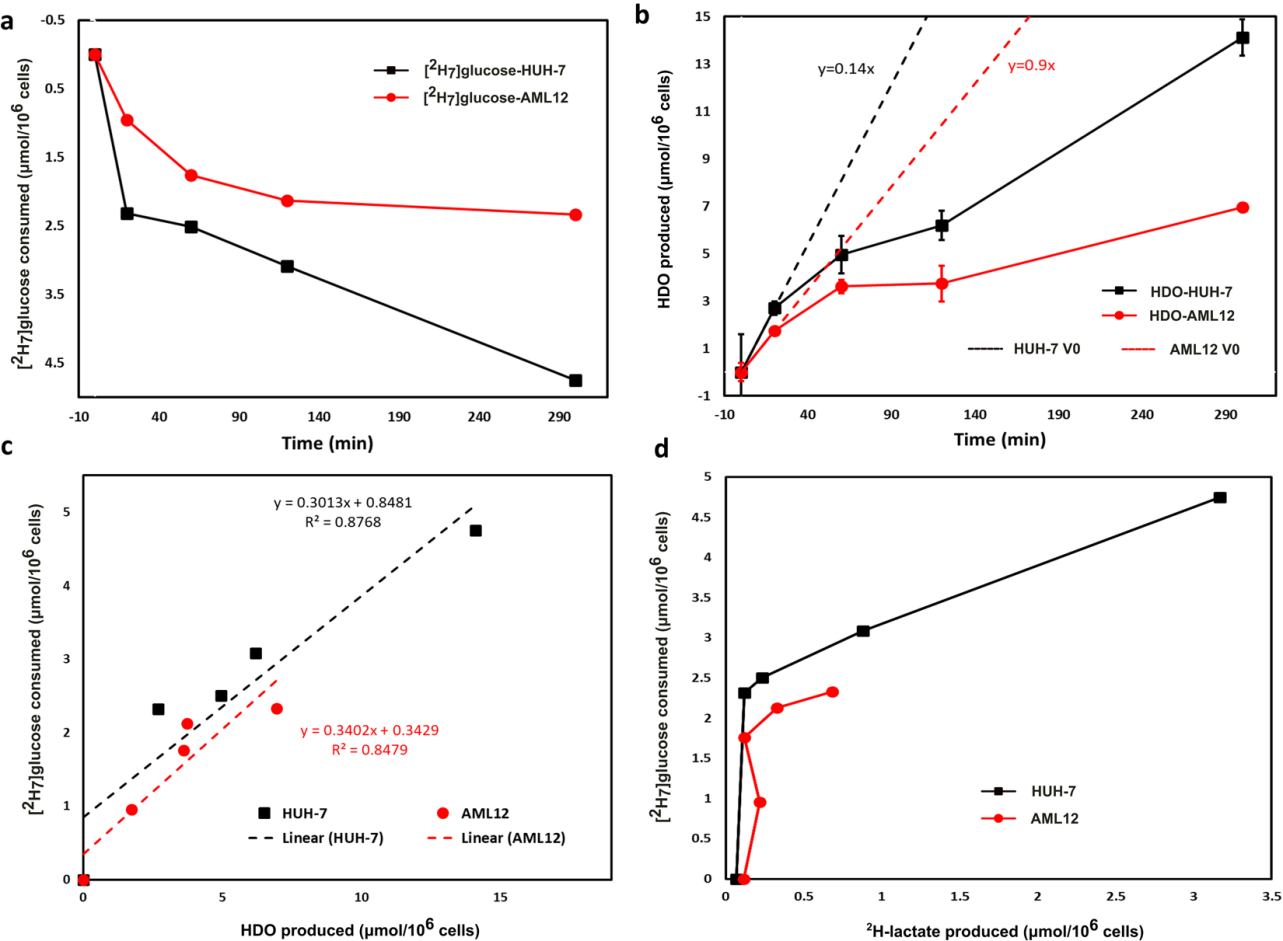
[^2^H_7_]glucose consumption and HDO production by HUH-7 and AML12 cells: Plot of (**a**) [^2^H_7_]glucose consumption and (**b**) HDO production by AML12 cells (n = 3, red circles) and HUH-7 cells (n = 4, black squares) during incubation in the DMEM with 5.5 mM [^2^H_7_]glucose. Panel (**c**) is the correlation between [^2^H_7_]glucose consumption and HDO production whereas panel (**d**) shows a lack of correlation between [^2^H_7_]glucose consumption and ^2^H-lactate production by HUH-7 (black squares) and AML12 cells (red circles). Results were calculated from ^2^H-NMR data of each of the cell media samples including a blank withdrawn at 0 min time point. Both graphs clearly indicate that the evolution of HDO and consumption of [^2^H_7_]glucose is very high in the case of HUH-7 cancer cell lines. Initial rates of HDO production (Panel b, dotted lines) should be robust estimators of glycolytic capacity. HDO production correlates well with glucose consumption in both the HUH-7 and AML12 cell lines. (Note: AML12 and HUH-7 cells has 3 (red circles) and 4 (black squares) biological replicates, respectively. [^2^H_7_]glucose consumption and HDO and ^2^H-lactate production were measured in the cell media withdrawn at 0 min, 20 min, 1 h, 2 h and 5 h time periods of AML12 and HUH-7 cells, using ^2^H-NMR. Error bars on some of plots are the size of the markers).

Pyrazine-D_4_ was used as an internal standard to quantify the HDO production and residual [^2^H_7_]glucose in each of the cell media samples of HUH-7 and AML12 cell lines. Peak areas of pyrazine-D_4_, HDO and residual [^2^H_7_]glucose were used to measure the concentrations of HDO and residual [^2^H_7_]glucose with respect to the 5 mM pyrazine-D_4_ internal standard. Cellular [^2^H_7_]glucose consumption is calculated from the residual [^2^H_7_]glucose in the cell media whereas HDO production was corrected for the natural abundance HDO (18.0 + /− 0.5 mM) in the cell media samples of each time points. Deuterium NMR is very linear with concentration and has been used in similar applications in the past^30^. Comparison of glucose consumption to HDO production from the perdeuterated substrate demonstrates the advantages of this tracer versus the [6,6-^2^H_2_]tracer. Initial consumption of the glucose is rapid, with a sharp drop in glucose concentration mirrored by increases in [HDO]. The data points at 0 and 20 minutes were fit to a linear model which yielded production rates of 0.14 μmols/min*10^6^ cells and 0.09 μmols/min*10^6^ for the HUH-7 and AML12 cell lines respectively (Fig. 6a,b). Glucose consumption correlated with HDO production well in both cell lines (Fig. 6c). Lactate production as measured by ^2^H detected NMR did not correlate well with glucose uptake in either cell line (Fig. 6d).

## Discussion

The ^1^H-NMR spectrum has a complicated pattern due to the very small geminal couplings (^2^*J*_*H-D*_) and vicinal couplings (^3^*J*_*H-H*_) of methyls of the partially deuterated lactate isotopomers (Fig. 2a). Since *J*_*H-D*_ is roughly 1/6^th^ of *J*_*H-H*_ (γD/γH = 1/6.48), most of the couplings appeared as a line broadening in ^2^H-decoupling-off ^1^H-NMR spectrum. To resolve the methyl signals of lactate, ^2^H-decoupling was implemented during the acquisition period to remove the small geminal couplings (^2^*J*_*H-D*_). The {^2^H}^1^H-NMR spectrum in Fig. 2b shows a distinguished pattern of the remaining vicinal couplings (^3^*J*_*H-H*_) of ~7.00 Hz for each of the methyl signals of deuterated lactate as well as unlabeled lactate^21^. Deuterium-induced perturbations of ^1^H-NMR chemical shifts (isotope shift) allows resolution of the lactate isotopomers. The extent of isotope shift of methyl proton increases in the order CH_3_ < CH_2_D < CHD_2_, which produces a measurable peak separation^31^. Utilizing the advantage of the isotope shifts we could resolve the lactate isotopomers in {^2^H}^1^H-NMR spectra (Fig. 2b). Metabolism of the [^2^H_7_]glucose produces lactate-CH_2_D and lactate-CHD_2_, which is also evident from the ^1^H-NMR spectrum (Fig. 2b). No trace of any lactate isotopomer was found in blank cell media (Fig. 3a). After 20 minutes of incubation of HUH-7 cells, efflux of unlabeled lactate signals can be observed, presumably derived from unlabeled glucose or pyruvate that remained in the HUH-7 cells (Fig. 3b). However, unlabeled C3-lactate is continuously produced even in the presence of perdeuterated glucose due to the loss of ^2^H from the lactate precursors during glycolysis, specifically through the action of phosphomannose isomerase (PMI), triose phosphate isomerase (TPI), and keto-enol tautomerization of pyruvate (Fig. 1). As incubation time proceeds, appearance of methyl-deuterated lactate dominated the total lactate methyl region, whereas the lactate-CH (4.12 ppm) signal pattern becomes more complicated due to multiple vicinal couplings (^3^*J*_*H-H*_) of lactate-CH with lactate-CH_3_, -CH_2_D, and -CHD_2_. The metabolic scheme of the conversion of exogenous [^2^H_7_]glucose to lactate *via* glycolysis shows that the lactate-C2 is not deuterated due to the action of enolase (Fig. 1). Hence, methyl signals for all of the lactate isotopomers are expected to be doublets in {^2^H}^1^H-NMR spectra, which can be clearly seen in panel B (Fig. 3b–d). These observations match those of de Feyter, *et. al*., but a detailed explanation of the multiplets facilitates our discussion of mass balance of the ^2^H label in relation to HDO production^21^.

Proton relaxation times of the lactate isotopomers increases incrementally with the number of deuterons (see Supplementary Fig. S3). The dipole-dipole interaction is the dominant mechanism in the proton T_1_ relaxation time, and introducing lower-γ nuclei lengthens the relaxation of nearby ^1^H nuclei. Therefore, the intramolecular dipole-dipole interactions between the spin ½ nuclei, *i.e*. proton-proton, dominate any *J*-quadrupolar cross-relaxation term. With this effect T_1_ relaxation times of the lactate isotopomers increase in the order of lactate-CH_3_, -CH_2_D and -CHD_2_. Concentration of each of the deuterated lactate isotopomers increases with incubation time. At the start of the cell culture incubation, lactate-CH_3_ derived from endogenous source was effluxed into the cell media. It is expected that HUH-7 cells have taken some time to transport and metabolize exogenous [^2^H_7_]glucose. Production of labeled lactate rapidly increases after 1 h of incubation time. The rate of production of lactate-CH_3_, lactate-CH_2_D and lactate-CHD_2_ was calculated using linear regression and found to be 0.0033, 0.0071, and 0.0.012 µmol/10^6^cells/min respectively. One of the key enzymes in glycolysis is aldolase (Fig. 1) which produces glyceraldehyde 3-phosphate (GA3P) and dihydroxyacetone phosphate (DHAP)^32,33^. GA3P proceeds down glycolysis and produces lactate-CHD_2_. DHAP converts into GA3P by activity of the triose phosphate isomerase (TPI) and then produces lactate-CH_2_D through the rest of glycolysis.

Relative percent contributions of lactate isotopomers to the efflux pool size demonstrate positive efflux thought the experiment (Fig. 5). At the 20 minute time point, contribution of lactate-CH_3_ was highest and labeled lactate contribution was low. Labeled lactate contribution rapidly increased whereas lactate-CH_3_ contribution decreased with incubation time. Labeled lactate contribution to the pool size was highest at 5 h and at this point lactate-CH_3_ has lowest contribution to the total pool size. It should be kept in mind that the lactate-CHD_2_ contribution to the pool size is always higher than that of the lactate-CH_2_D. Theoretically, the relative concentration and production rate of the lactate-CHD_2_ and lactate-CH_2_D should be equal, but we found differential concentration of the lactate isotopomers from [^2^H_7_]glucose. Total glycolytic rate is not affected by perdeuteration in heart tissue^34^, though we cannot firmly discount possible effects on total rate in these experiments.

Ben-Yoseph *et. al*. found a significant loss of ^2^H atoms from C1 and C6 of the [1-^2^H]glucose and [6,6-^2^H_2_]glucose respectively. Loss of ^2^H from C6 of glucose was mainly due to catalytic action of pyruvate kinase (PK) whereas loss of ^2^H from C1 of glucose was due to both PK and PMI (Fig. 1)^35^. De Feyter *et. al*. also showed that the loss of ^2^H atoms for ^2^H-lactate was ~8%, while using [6,6-^2^H_2_]glucose as a tracer^21^. The rate of ^2^H loss from lactate-CH_2_D and lactate-CHD_2_ precursors could be different due to deuterium secondary isotope effects; typical isotope effects may result in 10–15% higher loss of ^2^H from lactate-CH_2_D precursor^36,37^. The presence of a ^2^H kinetic isotope effect at TPI, results in more ^2^H liberation from the DHAP^38,39^. Due to these various factors involved in the metabolism of [^2^H_7_]glucose, a small amount of lactate-CH_3_ was continuously effluxed into the cell media from HUH-7 cells (Fig. 4). Pentose phosphate pathway (PPP) is another possible source of diversion of ^2^H from C1 of [^2^H_7_]glucose derived intermediates (Fig. 1)^36,40^. However, there was no measurable evidence of significant PPP flux in HUH-7 cells.

HDO will be produced by triose phosphate isomerase (TPI) from the C2 position of GA3P and DHAP, within the course of direct metabolism of one equivalent of [^2^H_7_]glucose to pyruvate (Fig. 1). HDO production from the C1/C2 positions can occur due to the interconversion of F6P and M6P *via* PMI^41^. Enolase activity on phospho-enol pyruvate (PEP) precursor and keto-enol tautomerization in pyruvate are also prominent sites of HDO production during glycolysis (Fig. 1)^21,32,33,35,40,42–49^. Apart from glycolysis, deuterium can also be lost to solvent water in the form of HDO in several reactions in the TCA cycle^50,51^.

Metabolism of [^2^H_7_]glucose produces an increase in the HDO signal that correlates with cellular glucose uptake in the HUH-7 cells, and a dramatic difference in HDO production has been observed between HUH-7 cancer cells and background precursor cells (AML12 cells) (Fig. 6). To prove the correlation of cellular [^2^H_7_]glucose uptake *versus* ^2^H-lactate and HDO production, we calculated the ^2^H mass balance at the 5 hour time point. Approximately 96% ^2^H atoms of [^2^H_7_]glucose were incorporated into ^2^H-lactate and HDO, strongly suggesting that the ^2^H-based Warburg metabolism dominates in HUH-7 cells (Table 1). [^2^H_7_]glucose uptake and HDO and ^2^H-lactate production were both much lower in AML12 cells. Glucose consumption correlates well with HDO production (Fig. 6c) indicating that HDO production is a robust analog to glucose uptake in normal and cancer cells. Lactate production measured by ^2^H-NMR does not serve as a surrogate for glucose uptake when using the perdeuterated substrate (Fig. 6d). On the contrary, the limited sensitivity of ^2^H detection causes lactate appearance to lag measured glucose consumption considerably. However, even with ^1^H detection of the lactate isotopomers, there is a noticeable, non-stoichiometric production of lactate from glucose in the initial 20 minute time period (see Supplementary Fig. S5). After the initial time point, lactate production scales in more logical ways versus the glucose consumption. We surmise that the limit of detection for lactate using both ^1^H and ^2^H NMR prevents rigorous mass balance at lower lactate levels. In contrast, since HDO production causes an increase in signal for the largest peak in the spectrum, it always results in a measurable effect. This suggests HDO production will serve as a more robust estimator of glycolytic flux than deuterated lactate production.

Based on parameters for oral^52^ and intravenous administration of glucose in humans and estimates of tumor geometry and cellularity^53^, HDO enrichment in the peritumoral area may reach several percent, sufficient to observe lengthening of ^1^H T_1_ due to less efficient dipole-dipole relaxation^54^. T_1_ weighted ^1^H imaging might therefore also be diagnostic, which would remove the need for purpose built ^2^H detection coils. A useful feature of our proposed tracer method versus methods based on TCA intermediate/lactate ratios is that it obviates the need to correct for ^2^H loss between the two species, reducing inter-subject variance and providing for easier extension to multiple tumor types.

From a practical point of view, {^2^H}^1^H-NMR experiments are easy to set up and require minimal time compared to direct ^2^H detection for ^2^H-lactate. Measuring the kinetics of the lactate isotopomer production from deuterated glucose by utilizing the more sensitive ^1^H-NMR experiment makes an easy method for measuring glycolytic rates. It is to be determined if this method could be translated for *in vivo* use. Lactate detection by ^1^H MRS is typically confounded by overlap of resonances derived from FAs. With a large FA signal the difference spectrum approach used here would have to be carefully implemented to prevent improper subtraction of the FA peaks and uncertainty in quantification of the ^2^H lactate. As compared to ^13^C based editing sequences, this method does not have to contend with a natural abundance correction. Also, the isotope shifts could potentially provide a more robust subtraction signal. The differential T_1_ of the lactate isotopomers is more difficult to take advantage of, as the T_1_ is lengthened upon ^2^H-enrichment. Standard T_1_ weighting would not provide contrast from the isotopomers. Specific choice of the delay time might allow the lactate-CH_3_ signal to be nulled which the ^2^H isotopomers remain using inversion recovery schemes.

In conclusion, Warburg metabolism is phenotypic of many cancers. Increased lactate production is a consequence of increased glucose consumption and reduced pyruvate dehydrogenase flux in cancer cells. Here we show that quantitation of HDO production may provide a simplified means for metabolic imaging of the deuterium signal specific to cancer cell glucose uptake, analogous to [^18^F]-FDG PET, with superior signal-to-noise compared to other metabolites like lactate and glutamate. As compared to earlier results with [6,6-^2^H_2_]glucose, this method retains the selectivity of lactate enrichment, but with the increased likelihood of HDO generation, amplifies the sensitivity of the experiment to increased glycolytic flux. Either technique can be implemented on preclinical or clinical hardware and may provide the basis for a safe, zero-dose method of imaging cancer glucose uptake and Warburg metabolism.

## Methods

### Cell line, chemicals, and media

HUH-7 hepatocellular carcinoma cells were received as a gift from Dr. Ralph Deberardinis at the Children’s Research Institute at University of Texas Southwestern Medical Center, Dallas, TX, USA. AML12 cell lines were purchased from the American Type Culture Collection (Manassas, VA). Pyrazine, Pyrazine-D_4_ and [1,2,3,4,5,6,6-^2^H_7_]-D-glucose ([^2^H_7_]glucose) were purchased from Sigma Aldrich, (St. Louis, MO, USA).

### Cell culture

HUH-7 and AML12 cell lines were cultured in a complete growth medium made up of Dulbecco’s Modified Eagle Medium (DMEM) with 10% v/v FBS, 10,000 units/mL penicillin, 10,000 µg/mL streptomycin, 25 µg/mL amphotericin B and 5.5 mM D-glucose. Cell lines were maintained at 37 °C in a 5% CO_2_ in air atmosphere in an air-jacketed incubator (Heracell Vios 160i, Thermo Scientific, Waltham, MA). Growth media was replaced every 3 days, and cells subcultured 1:5 at 80% confluence. Five 100 mm OD (56.7 cm^2^ culture area) cell culture plates containing 15 million HUH-7 or AML12 cells (total) at 70–80% confluence were washed once with warm PBS and incubated with 4 mL each of phenol red-free DMEM with 5.5 mM [^2^H_7_]-glucose for 5 h, withdrawing 100 µL aliquots at 20 min, 1 h, 2 h, and 5 h.

### Sample preparation

No extraction was performed on the cell media samples. Pyrazine and pyrazine-D_4_ were mixed to achieve 5 mM concentration of each of the standards in each cell media samples of HUH-7 and AML12 cells. Pyrazine and pyrazine-D_4_ served as an internal standard to quantify the lactate isotopomers from ^1^H-NMR data and HDO as well as residual [^2^H_7_]glucose from ^2^H-NMR data respectively. 40 µL of cell medium was transferred into 1.7 mm NMR tubes for NMR analysis.

### ^1^H-NMR spectroscopy

All NMR spectra were recorded on a 600.23 MHz NMR, equipped with a 1.7 mm TCI CryoProbe and Avance Neo Console (Bruker Biospin). All NMR experiments were set up in the lock off followed by sweep off mode. ^2^H-decoupling off and on ^1^H-NMR spectra were acquired with a sequence that alternated decoupling with each scan. Relaxation delay (d1) of 1.5 s and acquisition time (AQ) of 2 s (3.5 s of recycling time, Tr) and a 90° pulse of 10.50 µs was used for acquisition of each of the spectra. The WET method containing selective pulses was applied on the strong water resonance^55^. Waltz16 ^2^H-decoupling sequence consists of a 90° pulse of 250 µs with decoupling power of 1.72 watts, producing a B_1_ of 1 kHz during the acquisition period. 13,157 complex data points were acquired with the spectral width of 11ppm. 128 scans were used to acquire each spectrum. 4 dummy scans were also used for equilibration of the spin states prior to acquisition. Modified T_1_ inversion recovery pulse sequence with ^2^H-decoupling on during the acquisition was used to measure the T_1_ of the lactate isotopomers and pyrazine standard. A relaxation delay (d1) of 50 s and inversion recovery delays (τ) of 0.001, 0.6, 0.9, 1.5, 2.0, 3.0, 6.0, 9.0, 14.0, 20.0, 30.0 and 40.0 s were used to determine the T_1_ of lactate isotopomers.

### ^2^H-NMR spectroscopy

The deuterium lock channel was used to acquire the ^1^H-decoupled ^2^H-NMR spectra at 92.13 MHz resonant frequency. A relaxation delay (d1) of 2 s and acquisition time (AQ) of 1 s (3 s of recycling time, Tr) with a 90° pulse was used to acquire each of the ^2^H-NMR spectra. 1086 complex data points were digitized with the spectral width of 11 ppm using 1024 scans for each of the 5 FIDs (5120 scans) for each of the samples. The T_1_ inversion recovery pulse sequence was used to calculate the T_1_ relaxation times of HDO and pyrazine-D_4_ standard. A relaxation delay (d1) of 5 s, acquisition time (AQ) of 1 s and inversion recovery delays (τ) of 0.001, 0.1, 0.4, 0.8, 1.6, and 3.2 s were used to acquire an array of FIDs to calculate the T_1_ of HDO and pyrazine-D_4_. All NMR experiments were carried out at the room temperature (25 °C).

### ^1^H-NMR data processing and quantification of lactate isotopomers

NMR data analysis was performed using MestReNova *v*14.0.1-23284 (Mestrelab Research S.L.). For processing of the ^1^H-NMR spectrum, the spectra were zero-filled to 32,768 points with an exponential window function of 0.3 Hz before the Fourier Transform (FT). Manual phase and automatic spline baseline correction were performed on each of the spectra.

^2^H-decoupled ^1^H-NMR spectra of each time point were used for the extraction of the areas of lactate isotopomers and pyrazine internal standard. The MestReNova line fitting tool was used to extract the peak areas of the slightly overlapped signals for quantification of the lactate isotopomers. Concentration of the lactate isotopomers were measured with the relative concentration of pyrazine (5 mM) in each sample. T_1_ relaxation times were also measured for each of the lactate isotopomers and pyrazine standard. A T_1_ correction factor was used to calculate the absolute concentrations of each of the lactate isotopomers^56^.

### ^2^H-NMR data processing and quantification of HDO and residual [^2^H_7_]glucose

^2^H-NMR spectra were processed with zero-filling of FID to 4096 points followed by exponential window function of 1 Hz before the Fourier Transform (FT). Each spectrum was manually phase corrected and followed by automatic spline baseline correction. Five FIDs were acquired for each of the samples and all five ^2^H-NMR spectra were aligned and summed-up to compensate for peak shifting due to magnetic field drift over the course of the experiment.

Concentrations of HDO and residual [^2^H_7_]glucose in the cell media were calculated using peak areas of the internal standard pyrazine-D_4_, HDO, and residual [^2^H_7_]glucose from ^2^H-NMR spectrum. Concentration of pyrazine-D_4_ (5 mM) was used to measure the concentration of HDO and residual [^2^H_7_]glucose, normalized to the number of deuterons responsible for corresponding resonances.

## Supplementary information

Supplementary Information.

## Data Availability

Data generated or analyzed during this study are included in this article and its Supplementary Information files. Raw data is deposited on the metabolomics workbench.
