# Supplementary material for: HDO production from [2H7]glucose Quantitatively Identifies Warburg Metabolism
Source: Sci Rep. 2020 Jun 1;10:8885. doi: 10.1038/s41598-020-65839-8 (PMC7264272; doi:10.1038/s41598-020-65839-8)
Supplement: Supplementary file 1 — Supplementary Information. [file 41598_2020_65839_MOESM1_ESM.pdf]

HDO production from [$^2H_7$]glucose Quantitatively Identifies Warburg Metabolism

Rohit Mahar[1], Patrick L. Donabedian[1], Matthew E. Merritt[1]

[1]Department of Biochemistry and Molecular Biology, College of Medicine, University of Florida, Gainesville, FL, USA, 32610.

Correspondence and requests for materials should be addressed to M.E.M. (email:matthewmerritt@ufl.edu)

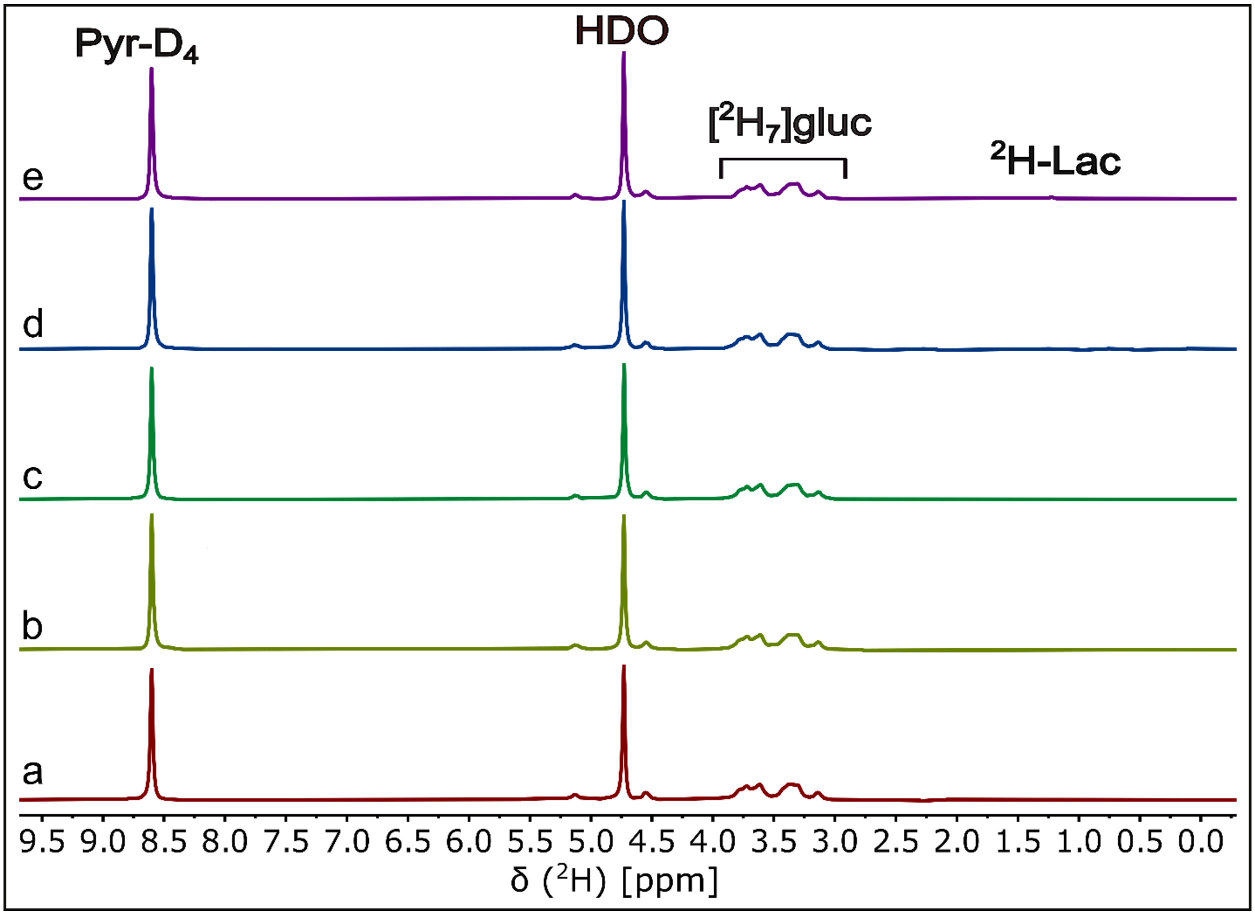

Supplementary Figure S1. ^{2}H-NMR spectra of the AML12 cell media at different time points: Stacked plot of the ^{1}H-decoupled ^{2}H-NMR spectra of the AML12 cell line incubated with 5.5 mM [$^2H_7$]glucose. Cell media withdrawn at (a) 0 min, (b) 20 min, (c) 1 h, (d) 2 h and (e) 5 h time points from cultures of AML12 cancer cells. Labeling on each peak in the (e) spectrum showing the resonances arise from the pyrazine-D_4, HDO, residual [$^2H_7$]glucose, and ^{2}H-lactate.

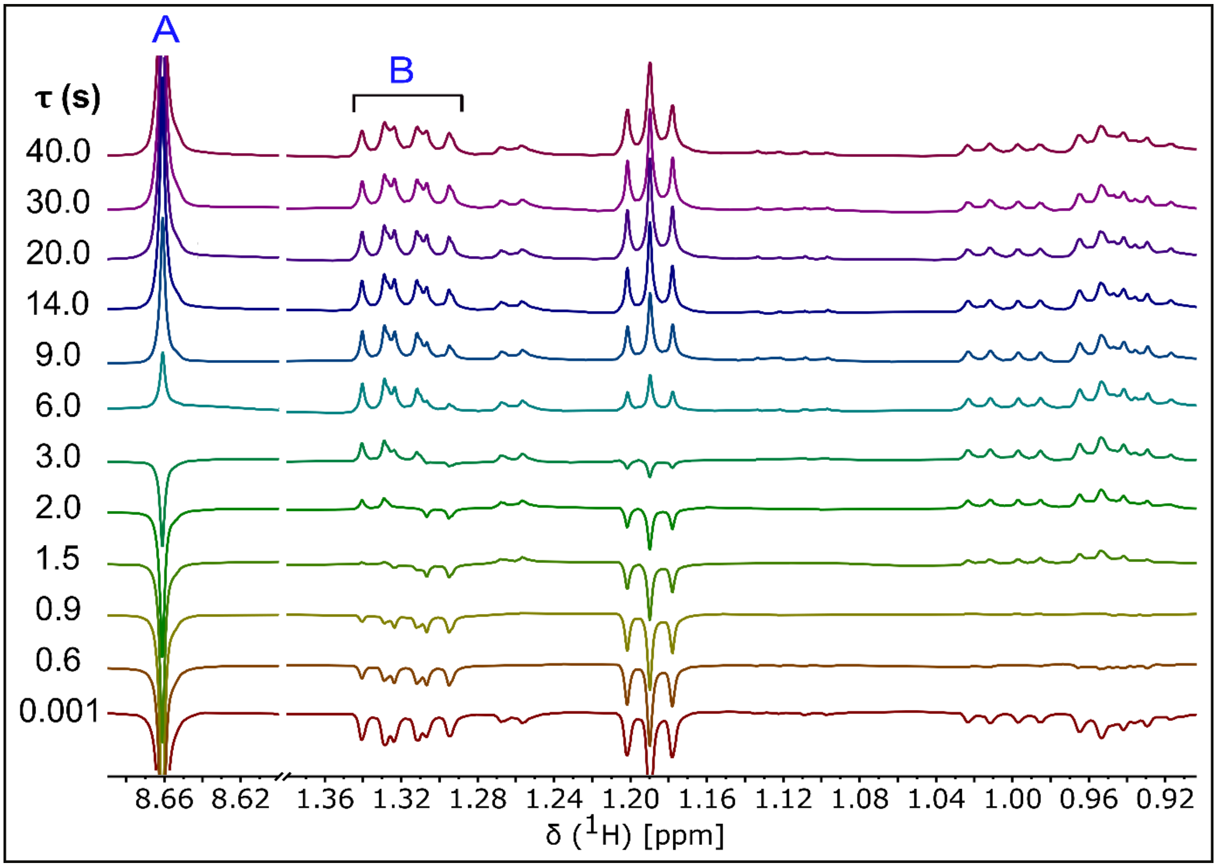

Supplementary Figure S2. Inversion recovery spectra of pyrazine and lactate isotopomers: Stacked plot of the T_1-inversion recovery ^{1}H-NMR spectra recorded with ^{2}H-decoupling during acquisition. Inversion recovery delays (τ) used to acquire the FIDs have been indicated in the front of respective spectrum. A and B marks represent the pyrazine and lactate isotopomers peak respectively.

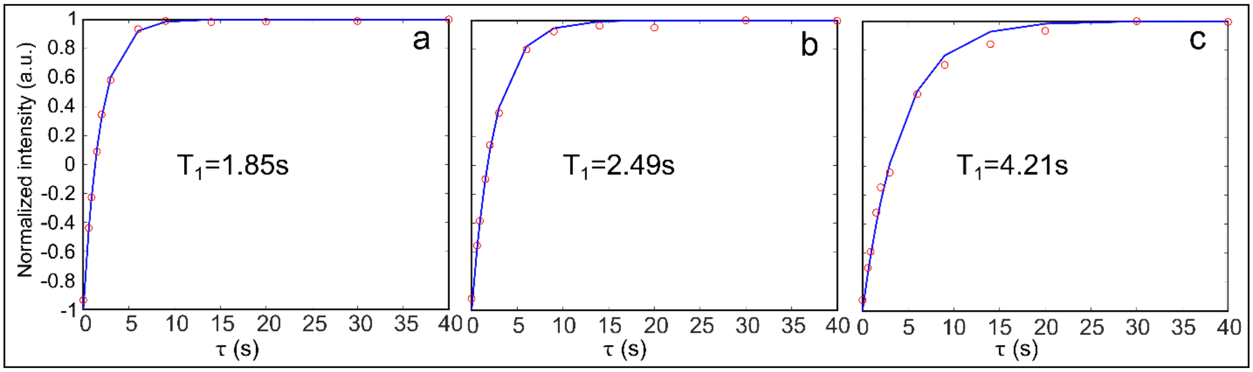

Supplementary Figure S3. Proton T_1 relaxation times of the lactate isotopomers: Longitudinal relaxation time (T_1) fitting curves and calculated T_1 relaxation times for (a) lac-CH_3, (b) lac-CH_2D and (c) lac-CHD_2 isotopomers. T_1 relaxation times were calculated using the normalized T_1-inversion recovery ^{1}H-NMR data and the standard inversion recovery equation.

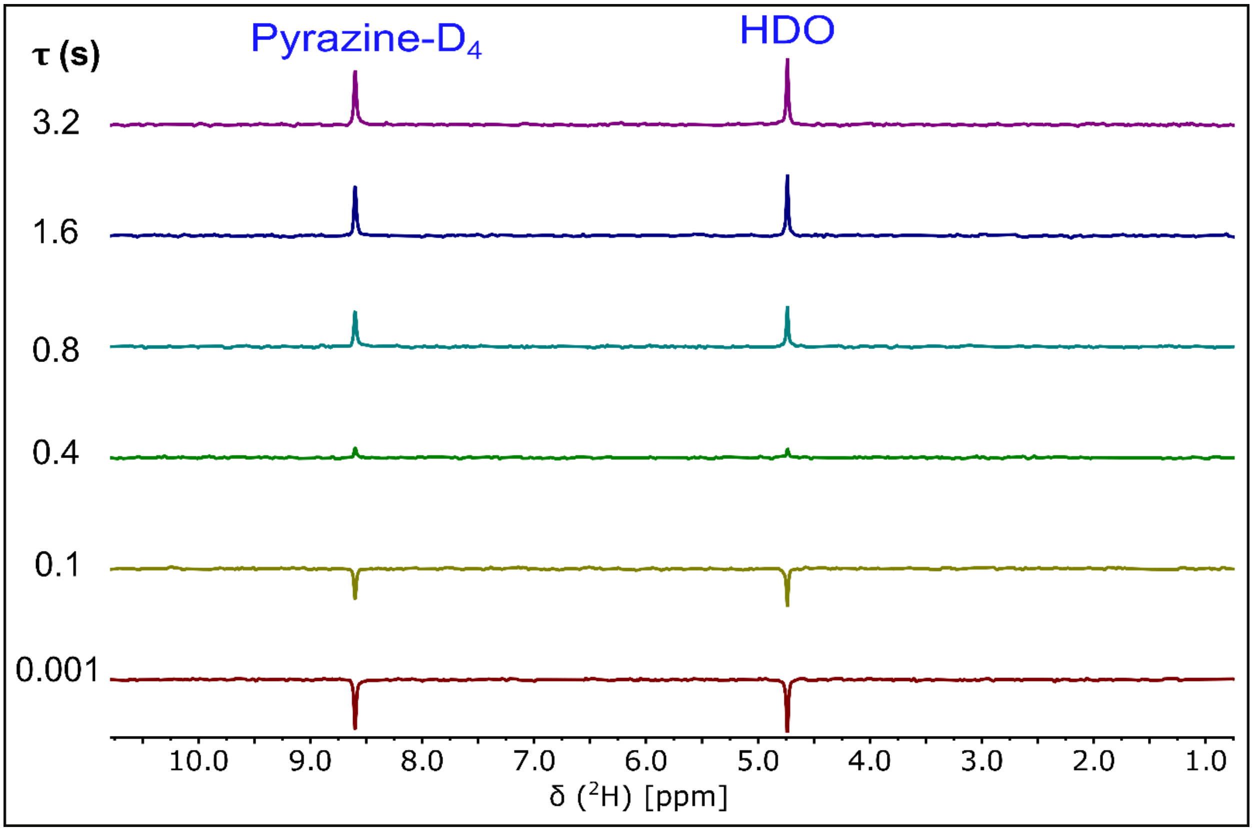

Supplementary Figure S4. Deuterium T_1-inversion recovery spectra: Stacked plot of the T_1-inversion recovery ^{2}H-NMR spectra, showing the pyrazine-D_4 and HDO resonances. Inversion recovery delays (τ) used to acquire the FIDs have been indicated in the front of respective spectrum. T_1 relaxation times were calculated for HDO and pyrazine-D_4 and found to be 0.49s and 0.45s respectively.

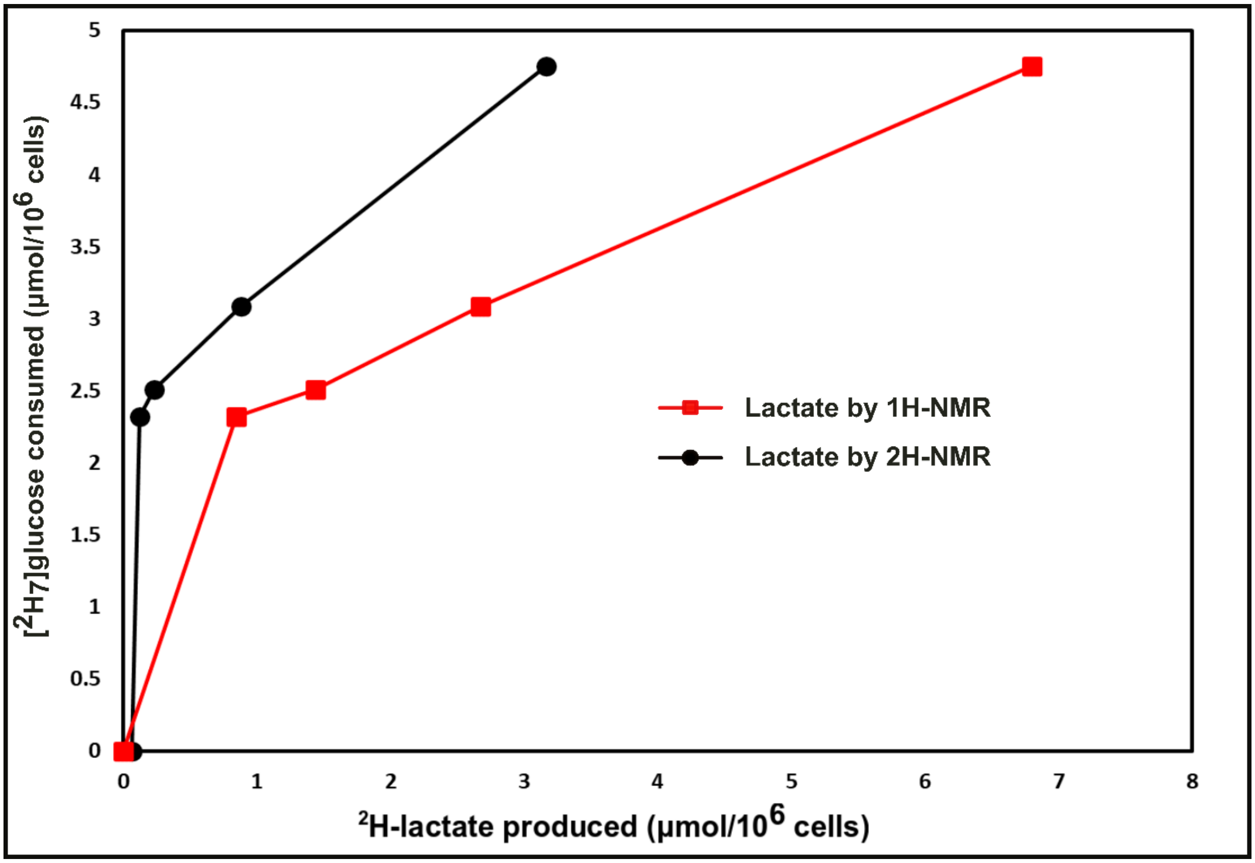

Supplementary Figure S5. Lactate production measured by ^{1}H and ^{2}H NMR: Plot of the correlation between [$^2H_7$]glucose consumption and lactate production from HUH-7 cells. Red circles and black squares showed the lactate concentration, measured by ^{1}H and ^{2}H NMR, respectively. The ^{1}H-NMR has very high sensitivity to measure the lactate isotopomers produced from [$^2H_7$]glucose.

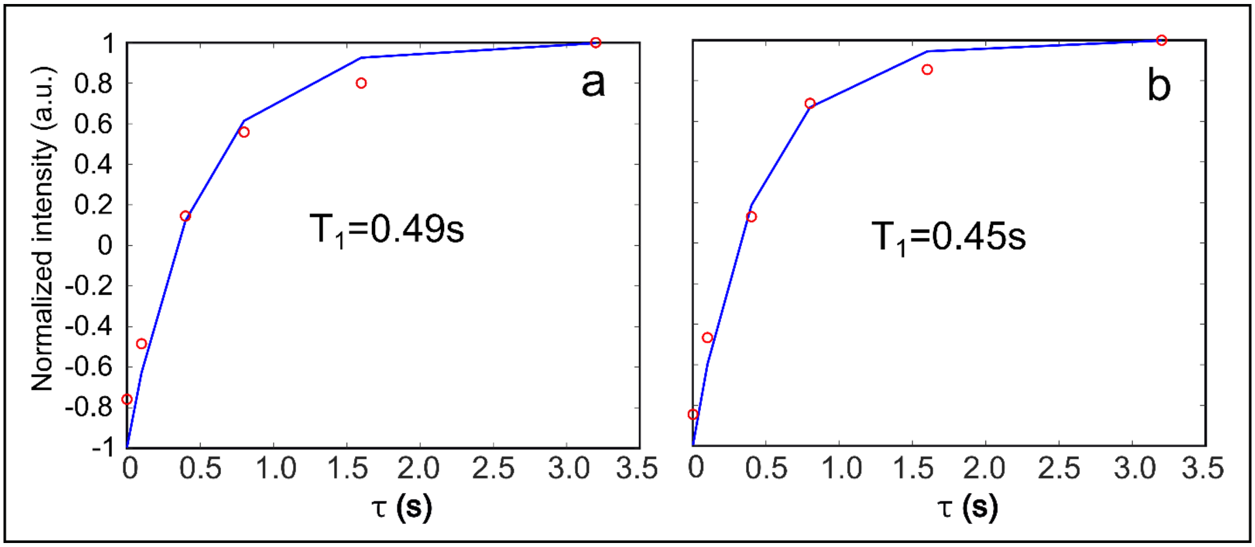

Supplementary Figure S6. Deuterium T_1 relaxation times of the HDO and pyrazine-D_4 isotopomers: Longitudinal relaxation time (T_1) fitting curves and calculated T_1 relaxation times for (a) HDO and (b) pyrazine-D_4. T_1 relaxation times were calculated using the normalized inversion recovery ^{2}H-NMR data and the standard inversion recovery equation.
